# Outcome and predictors of response to vagus nerve stimulation for drug-resistant epilepsy: a retrospective cohort study

**DOI:** 10.1007/s10143-025-03839-w

**Published:** 2025-10-03

**Authors:** Robert Crutcher, David Horvat, Nicholas Lehman, Yitao Ma

**Affiliations:** 1 Department of Neurology, School of Graduate Medical Education, Mayo Clinic, Jacksonville, FL USA; 2 Department of Neurology, Uniformed Services University of the Health Sciences, Bethesda, MD USA; 3 Department of Neurology, Walter Reed National Military Medical Center, Bethesda, MD USA

**Keywords:** Vagus nerve stimulation, Drug-resistant epilepsy, Generalized epilepsy, Outcome assessment, Responder analysis

## Abstract

Vagus nerve stimulation (VNS) has been used as an adjunctive therapy for patients with drug-resistant epilepsy (DRE) for decades. Nonetheless, the predictors of response to VNS remain inadequately characterized. We aimed to find factors associated with responder status. We retrospectively reviewed records of patients who received VNS for DRE in our medical center between 2000 and 2023. Preoperative data and VNS parameters were analyzed with univariate and multivariate analysis to identify predictors associated with responder status. We used receiver operating characteristic (ROC) curves to select possible related factors and the cutoff values. The overall responder rate was 35.8%. Individuals with generalized epilepsy demonstrated significantly higher responder rates (86%) compared to those with focal (29%) or combined epilepsy (28%). Univariate analysis indicated that an epilepsy duration of < 5 years at the time of VNS implantation (*p* = 0.021) and a diagnosis of generalized epilepsy (*p* = 0.016) were associated with a favorable response. A multivariate logistic regression analysis confirmed these findings. There was no correlation between age at implantation, prior epilepsy surgery, VNS parameters, such as output current and duty cycle, and the responder status. An epilepsy duration of < 5 years at VNS implantation and generalized epilepsy are independent predictors of responder status to VNS. Future long-term, randomized controlled trials with larger cohorts are needed to better evaluate these critical variables and their association with VNS outcomes.

## Introduction

Epilepsy affects approximately 60 million people worldwide, making it one of the most common neurological disorders [1]. Approximately 30% of all epilepsy patients develop drug-resistant epilepsy (DRE), defined as ongoing seizures despite adequate trials of two well-tolerated, appropriately chosen anti-seizure medicines (ASMs) [2]. In well-chosen candidates with DRE, surgical resection of a structural epileptogenic focus may be curative. Patients who are not surgical candidates may pursue other treatment modalities. One such intervention is vagus nerve stimulation (VNS), a neuromodulation technique that has gained recognition for its effectiveness in reducing seizure burden since its approval in Europe in 1994. VNS was approved in the United States as an add-on therapy to treat focal seizures in adults and children 4 years and older [3]. Over the past two decades, numerous studies have demonstrated that VNS can reduce seizure frequency by more than 50% in nearly 40–70% of patients [4–7]. There are also additional benefits of improved mood, cognitive function, and quality of life (QOL) [8–11].

Vagus nerve stimulation (VNS) entails a generator implanted in the chest and a lead attached to the vagus nerve (usually the left side) to provide chronic intermittent electrical stimulation to regulate brain activity. The precise mechanisms of action of VNS are not fully understood but are thought to involve modulation of thalamocortical pathways, regulation of neurotransmitters including glutamate, norepinephrine, and gamma-aminobutyric acid (GABA), as well as anti-inflammatory effects [12, 13]. Despite decades of clinical use, the predictors of VNS response remain poorly defined and continue to be a subject of debate [14–17]. Given the heterogeneity of patients undergoing evaluation of VNS implantation, currently there is no consensus on clinical predictors of VNS response. Using advanced neuroimaging techniques, Berger et al. found that a higher integrity of locus coeruleus-hippocampus connections and the structural integrity of thalamocortical tracts may be linked to the responder status of VNS therapy [18, 19]. However, these techniques are still under development and not widely available in all centers. Accurate identification of VNS responders is essential to reduce surgical risks and to guarantee efficient deployment of healthcare resources. The goal of our study is to identify potential predictors that may affect VNS responder status during long-term follow-up.

## Materials and methods

This is a retrospective cohort study of patients of all ages who received VNS for DRE at Walter Reed National Military Medical Center (WRNMMC) from January 2000 to December 2023. Patients with VNS for the treatment of diseases other than epilepsy were excluded. This study was approved by the Institutional Review Board at WRNMMC. We reviewed the records of 55 consecutive patients. Two records were excluded due to loss of follow-up right after VNS implantation. The VNS implantations were performed by staff neurosurgeons according to standard procedures [20]. After 2015, all patients received new models with automatic stimulation (AutoStim) during battery replacement. All patients in this study suffered from refractory epilepsy of different etiology and epilepsy syndromes. Patient demographics were collected using electronic medical records. Clinical neurology and neurosurgery notes were examined to gather variables associated with VNS therapy, including epilepsy type (categorized as focal, generalized, or combined), seizure etiology, age at seizure onset, age at implantation and study, neuroimaging findings, electroencephalography (EEG) results, seizure frequency, number of antiseizure medications (ASMs), and VNS parameters. Epilepsy types and syndromes were classified according to the International League Against Epilepsy criteria [12]. Responders were defined as more than a 50% reduction in seizure frequency following initiating VNS therapy at the most recent follow-up visit. Provoked seizures or seizure clusters from factors such as infection or medication noncompliance were not counted as average seizure frequency. ASMs were adjusted according to each patient’s clinical presentation and the preferences of providers, patients, and caregivers. Additional ASMs were introduced as needed to control severe seizures and enhance patient safety.

### Statistical analysis

All statistical analyses were performed using R Statistical Software (v4.4.0). We found that all continuous variables, including age at VNS implantation, output current, and duty cycle, did not follow a normal distribution; therefore, we used the median, minimum, and maximum to describe these variables. For each continuous variable (age at VNS implantation in years, epilepsy duration in years, output current in mA, and duty cycle in %), we assessed differences in VNS responder and non-responder groups using the Wilcoxon rank-sum test. For each continuous variable, we produced receiver operating characteristic (ROC) curves for VNS response using the method described by Hsieh and Turnbull [21]. We calculated the area under the ROC curve and identified an optimal threshold for discrimination between responders and non-responders according to the Youden Index [22]. For categorical variables including epilepsy type (focal, generalized, or combined), resective surgery prior to VNS, and subgroups of epilepsy duration, age at VNS implantation, output current, and duty cycle as identified above, we reported proportions of responders in each group. We used Fisher’s exact test to determine whether the relationship between these variables and VNS response was statistically significant. *P* < 0.05 was considered significant.

To adjust for potential confounding factors, we performed binary logistic regression analyses using Firth’s bias-reduced logistic regression via the logistf package in R (1.26.0) [23]. Odds ratios (ORs), 95% confidence intervals (CIs), and p-values for each variable were calculated to describe the risk factors associated with response.

## Results

Clinical and demographic information for our sample population is shown in Table 1. Among the 53 patients included in this study, the median age at the time of the study was 28 years, with ages ranging from 8 to 91 years. The median age at VNS implantation was 18 years, ranging from 2 to 83 years. Forty patients (75.5%) had childhood-onset epilepsy. The average duration of VNS therapy was 11.2 years, ranging from 0.25 to 24 years. There were 22 patients who underwent VNS therapy for over a decade. Overall, a total of 19 patients (35.8%) were responders, and 17 of them (89.5%) had childhood-onset epilepsy. Twenty-eight patients (52.8%) were diagnosed with developmental epileptic encephalopathy, including 14 patients (26.4%) with Lennox-Gastaut Syndrome (LGS). Ten patients (18.9%) had a neuronal migration disorder. Over the 24 years, twelve patients discontinued VNS therapy, including four deaths, three from lack of efficacy, four due to complications from the VNS implantation procedure, and one due to exercise intolerance. Twenty-two individuals (41.5%) experienced less than a 50% reduction in seizure frequency and were classified as non-responders. Nonetheless, they opted to continue with VNS therapy, as it offered modest benefits and caused minimal side effects. The retention rate was 77.4%. Thirty-three patients received VNS models with automatic stimulation (AutoStim). Seventeen of them remained to be responders, while 16 remained to be non-responders. Overall, the new models showed no significant additional benefit in seizure control.

**Table 1:** Clinical and demographic information for all included patients

Variable	*N* (%) or Median (Min, Max)
Sex	
Male	30 (56.7%)
Female	23 (43.4%)
Age Group	
Children	40 (75.5%)
Adults	13 (24.5%)
Age at study (Years)	
Median (Min, Max)	28 (8, 91)
Age at VNS Implant (Years)	
Median (Min, Max)	18 (2, 83)
Duration of Therapy (Years)	
Median (Min, Max)	11.2 (0.25, 24)
Epilepsy Type	
Focal	28 (52.8%)
Generalized	7 (13.2%)
Combined	18 (34.0%)
Surgery Prior to VNS	
Yes	5 (9.4%)
No	48 (90.6%)
Duration of Epilepsy (Years)	
Median (Min, Max)	9 (0.25, 34)
VNS Response	
≥ 50% Seizure Reduction	19 (35.8%)
< 50% Seizure Reduction	34 (64.2%)
Output Current (mA)	
Median (Min, Max)	1.75 (0.25, 2.5)
Duty Cycle (%)	
Median (Min, Max)	16 (5, 49)
EEG findings	
Focal	47 (88.7%)
Generalized Spike & Wave	17 (32.1%)
Epilepsy Etiology	
Unknown	31 (58.5%)
Developmental Epileptic Encephalopathy	28 (52.8%)
Neuronal migration disorders	10 (18.9%)
Infection	3 (5.7%)
Tuberous sclerosis complex	1 (1.9%)
Genetic/metabolic disorders	9 (17.0%)
Vascular lesion/tumor	1 (1.9%)
Traumatic brain injury	1 (1.9%)

Abbreviations: VNS, vagus nerve stimulation; EEG, electroencephalogram; MRI, magnetic resonance imaging

**Fig. 1:**
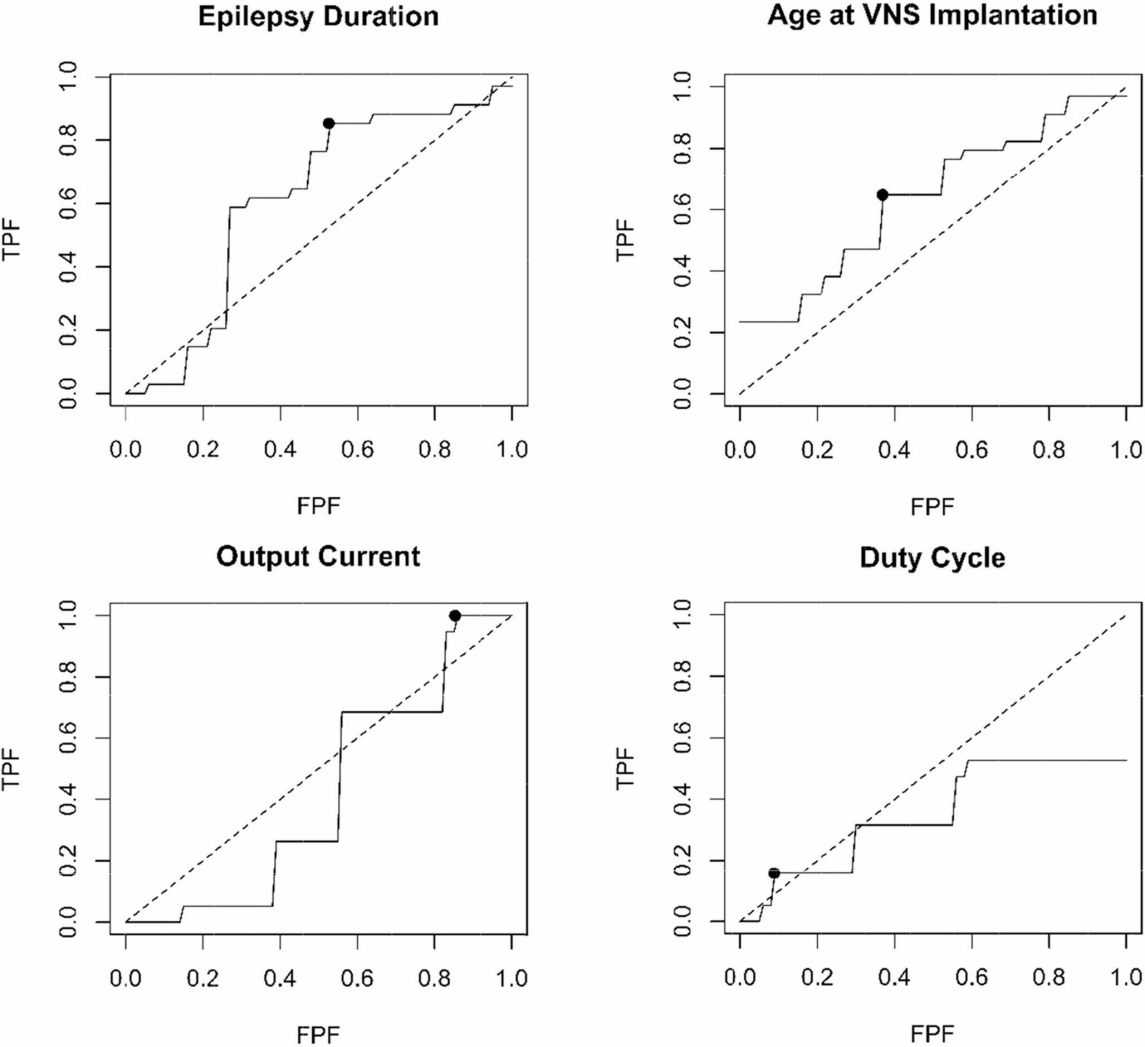
The receiver operating characteristics (ROC) of continuous variables. TPF = True Positive Fraction, equivalent to sensitivity. FPF = False Positive Fraction, equivalent to 1 – Specificity. The solid line indicates the ROC curve; the dashed line indicates the diagonal (signifies random chance); the point indicates the optimum threshold by the Youden Index (see Table 3)

We examined the continuous variables and their associations with VNS outcomes, including age at VNS implantation, epilepsy duration, output current, and duty cycle. None were statistically significant by the Wilcoxon rank-sum test (Table 2). The median epilepsy duration before VNS placement in years was lower among VNS responders (5 years) vs. non-responders (11.5 years); however, the overlap in the distribution between these groups was substantial (1–34 years for responders, 0.25-30 years for non-responders), and the difference was not statistically significant (*p* = 0.13) (Table 2). We then plotted receiver operating characteristics (ROC) curves for each variable to calculate optimum threshold values for each variable and the sensitivity and specificity they provide (Fig. 1). We found that epilepsy duration < 5 years identified responders in our population with 85% sensitivity and 47% specificity (Table 3). 100% of responders had output current > 1 mA; however, this cutoff was only 15% specific. Conversely, a duty cycle > 25% was 91% specific for VNS response in our population but only 16% sensitive. The ROC curves also suggested that age at VNS implantation < 18 years might affect the response. We found that the area under the ROC curve (AUC) for epilepsy duration and age at implantation were higher than those of the VNS parameters, although we noted that the 95% confidence interval (CI) for every AUC contained 0.5, the value associated with random chance.

**Table 2:** Association of continuous variables and VNS responses

Variable	Responders Median (Min, Max)	Non-Responders Median (Min, Max)	*P*-value
Age at VNS Implantation (Years)	17 (3.3, 42.8)	21 (2, 79)	0.081
Epilepsy Duration (Years)	5 (1, 34)	11.5 (0.25, 30)	0.13
Output Current (mA)	1.75 (1.25, 2.5)	1.75 (0.25, 2.5)	0.99
Duty Cycle (%)	12 (5, 41)	16 (10, 49)	0.69

**Table 3:** Receiver operating characteristics (ROC) of continuous variables and identification of threshold values

Variable	AUC (95% CI)	Optimum Threshold	Sensitivity	Specificity
Age at VNS Implantation (Years)	0.646 (0.490, 0.796)	< 18 Years	65%	63%
Epilepsy Duration (Years)	0.626 (0.433, 0.798)	< 5 Years	85%	47%
Output Current (mA)	0.502 (0.348, 0.653)	> 1 mA	100%	15%
Duty Cycle (%)	0.467 (0.387, 0.639)	> 25%	16%	91%

 We then did a binary univariate analysis of categorical variables, including epilepsy types and surgery before VNS, and continuous variables based on threshold values from the ROC curves to try to identify potential predictors of a good VNS response (Table 4). 64% of patients who had an epilepsy duration of < 5 years were responders compared to 26% with longer epilepsy duration, a difference that was statistically significant (p = 0.021). There were 5 patients (9%) who had epilepsy surgery prior to VNS placement. It is interesting that none of the patients with prior surgical resections were classified as responders to VNS (and all had epilepsy duration for more than 5 years at VNS placement) (Table 4).

**Table 4:** Univariate analysis of categorical variables on the VNS response

Variable	Responders (*N* = 19)	Non-Responders (*N* = 34)	*P*-value
Epilepsy Type			
Focal	8 (29%)	20 (71%)	**0.016**
Generalized	6 (86%)	1 (14%)	
Combined	5 (28%)	13 (72%)	
Surgery Before VNS			
Yes	0 (0%)	5 (100%)	0.15
No	19 (40%)	29 (60%)	
Epilepsy duration			
<5 years	9 (64%)	5 (36%)	**0.021**
>=5 years	10 (26%)	29 (74%)	
Age at VNS Implant			
Adult (> = 18 years)	7 (24%)	22 (76%)	0.084
Pediatric (< 18 years)	12 (50%)	12 (50%)	
Duty Cycle			
<=25%	16 (34%)	31 (64%)	0.65
>25%	3 (50%)	3 (50%)	
Output Current			
<=1 mA	0 (0%)	5 (100%)	0.15
>1 mA	19 (40%)	29 (60%)	

Even though VNS was only approved to treat focal epilepsy in the US, it has been used off-label for patients with generalized epilepsy. We included two patients with refractory Juvenile Myoclonic Epilepsy (JME) and one patient with refractory Juvenile Absence Epilepsy (JAE), two with epilepsy with myoclonic atonic seizures (EMAtS) and two patients with epilepsy with generalized tonic-clonic seizures alone (EGTCS). Their EEGs showed generalized spike and wave complexes. Magnetic resonance imaging (MRI) of the brain demonstrated no focal lesions. All of them except one were responders. The non-responder reported magnets helped abort myoclonus clusters. VNS helped decrease all seizure types in these patients, including GTCs, absence seizures, and atonic seizures. The responder rate of patients with generalized epilepsy (86%) was significantly higher than that of patients with focal epilepsy (29%) or combined epilepsy (28%) (Table 4). The p-value was 0.016, indicating generalized epilepsy is a predictor for good response. 

 VNS parameters, including a duty cycle >25% and an output current>1 mA, did not prove to be significant predictors for VNS response (Table 4). While some evidence in the literature suggests that increased duty cycles could significantly increase efficacy [24], we did not find this to be the case in our population (p = 0.65). Rapid cycling, with off-time ≤1.1 min, was employed in six patients who did not have a significant seizure reduction after reaching the standard target settings. Three of them with duty cycles of 41%, 35%, and 29% were responders, while another three patients with duty cycles of 41%, 49%, and 35% were non-responders. There was no significant difference in output current between responders and non-responders (p=0.15).

 In a multivariate logistic regression analysis (Table 5), we found that epilepsy duration of < 5 years was a strong independent predictor for successful VNS response (OR = 7.23, p = 0.01) after adjusting for epilepsy type, epilepsy surgery prior to VNS, age at VNS implantation, duty cycle, and output current. Similarly, generalized epilepsy was also a predictor for a good response compared to focal epilepsy, combined epilepsy, and other covariates (OR = 8.62, p = 0.027). The effects of epilepsy duration of < 5 years and generalized epilepsy were clearly independent of one another, as only 1 of 7 (14%) generalized epilepsy patients had epilepsy duration of < 5 years at VNS implantation, compared to 6 of 22 (27%) focal epilepsy patients and 7 of 11 (64%) combined epilepsy patients.

**Table 5:** Multivariate logistic regression of predictors for the VNS response

Variable	Odds Ratio	95% CI	*P*-value
Epilepsy duration < 5 years	7.23	1.56, 46.9	**0.010**
Epilepsy Type			
Focal	-		-
Generalized	8.62	1.26, 101	**0.027**
Combined	0.429	0.066, 2.08	0.30
Surgery Before VNS	0.47	0.003, 5.60	0.60
Age < 18 years at Implant	1.49	0.294, 7.29	0.62
Duty Cycle > 25%	0.583	0.067, 4.36	0.60
Output Current > 1 mA	7.29	0.471, 1250	0.18

Higher odds ratios are indicative of higher odds of VNS response. Boldface type indicates statistical significance

## Discussion

We presented the outcome data of patients with DRE who were treated with VNS in the past 24 years in our center. Among the 53 patients included in this study, 19 (35.8%) were responders. The efficacy was sustained over a period of up to 24 years from the initiation of the therapy. Our study indicated that epilepsy duration of < 5 years and generalized epilepsy were independent predictors of a favorable response to VNS in terms of seizure frequency reduction. No correlation was identified between the age of VNS implantation, prior epilepsy surgery, and VNS settings, including duty cycle and output current, with the outcome

In published studies, responder rates for implanted VNS vary from 22 to 74.3% [3]. Efficacy appears to increase over time. The response rate observed in our study is comparatively lower than that reported in other studies. This may relate to different patient populations, epilepsy types, and interrater variability. We included 24 patients (45.2%) with LGS and neuronal migration disorders. Previous studies indicated VNS might not work well for these groups of patients [7, 25, 26]. This is a long-term study with a median therapy duration of 11.2 years. Patients tolerated VNS well for up to 24 years without significant side effects. Both responders and partial responders, who experienced less than 50% seizure reduction, continued VNS therapy, indicating that VNS is a very well-tolerated treatment option.

The study revealed that epilepsy duration of < 5 years is a strong independent predictor of responder status. There have been inconsistencies in available data regarding epilepsy duration and clinical outcome following VNS treatment. Multiple studies have demonstrated that the clinical outcome of VNS in patients with a shorter epilepsy duration was significantly superior to those with a long seizure history [14–16, 26, 27]. This is especially true in younger children with a brief history of epilepsy whose VNS therapy is particularly effective [28]. Other studies found reduced seizure burden and improved QOL were more often observed in patients implanted at a younger age and with a shorter duration of epilepsy [29, 30]. In contrast, Labar has indicated that an extended epilepsy duration correlated with improved VNS outcome [31]. Chrastina et al. stated that extended epilepsy duration did not adversely affect the efficacy of VNS [32]. These studies suggest that VNS can provide benefits to epilepsy patients across various stages of the disease. However, extended periods of epilepsy may cause irreversible damage to the central nervous system, which could influence patients’ response to VNS. Chronic, uncontrolled seizures can profoundly impact the QOL of both children and adults. While VNS is primarily a palliative treatment, offering it to the right candidates early on may help ensure they gain the greatest benefit from the procedure.

Seven patients with refractory generalized epilepsy received VNS therapy in our center. For patients with generalized DRE, surgical options are limited, and neurostimulation, including VNS, can be a favored therapeutic option. The mechanism of action of VNS supports its use for generalized epilepsy. Studies indicate that patients with generalized epilepsy experience progressive dysfunction of thalamic neurons [33]. VNS works by enabling higher-order neurons from vagal afferents to synapse at the nucleus tractus solitarius, which then projects to the thalamic nuclei, limbic system, and thalamocortical circuit. This interaction helps regulate the network’s activity, disrupt the epileptogenic pathways, and ultimately improve seizure control. Jaafar et al. reported that five out of six pediatric patients with EMAtS who underwent VNS implantation demonstrated significant improvement in seizure frequency; three became seizure-free, and four showed positive neuropsychological outcomes [34]. A study by Ny and Devinsky showed patients with generalized epilepsy responded better than those with focal epilepsy [35]. In Europe, VNS is approved for all seizure types, whereas in the US, it is only approved for focal epilepsy [3]. A comparative study indicated that VNS was significantly more efficacious in focal epilepsy compared to patients with symptomatic generalized epilepsy or idiopathic generalized epilepsy groups [36]. Englot et al. observed that patients with predominantly focal seizures, especially simple-partial seizures and auras, posttraumatic epilepsy, and tuberous sclerosis were strong indicators of a positive response to the treatment [15]. Lagae et al. verified that the VNS outcome did not significantly differ between generalized and focal epilepsies [17]. A study by Kostov et al. found that although epilepsy type was not a predictor of being a responder, patients with generalized epilepsy were more likely to be seizure-free at the last observation [37]. Our study, despite involving a limited number of patients, indicated that VNS showed greater clinical efficacy in patients with generalized epilepsy compared to those with focal epilepsy. Notably, almost all patients with generalized epilepsy responded positively to VNS in long-term follow-ups. Our result suggests that VNS could be a valuable therapeutic option for this group of patients. The responder rate of generalized epilepsy in our study exceeds that of prior studies [16, 35]. This may be the result of chronic neuromodulation from a more extended therapeutic duration. The natural disease progression of generalized epilepsy over such a prolonged period may serve as a confounding factor. Future randomized controlled trials (RCTs) with a larger cohort are essential to validate this finding.

This study has limitations, including a limited sample size, which may constrain the generalizability of its findings to a wider population. Additionally, as with all retrospective record review studies, challenges arise from incomplete data, recall bias, selection bias, and the presence of confounding variables. Our study relied on self-reports of seizure frequency provided by patients and their caregivers, which introduced the potential of either underestimating or overestimating the actual occurrence of seizures. Most patients had changes in ASMs and VNS parameters during their therapy with VNS, which may confound their outcomes. In addition, we did not assess the impact of VNS on cognitive development and QOL, both of which are significant aspects of neuromodulation. Finally, our population of military beneficiaries may differ from a typical civilian population in terms of social determinants of health. While the impact of health insurance on practice patterns is mitigated in the military health system, frequent moves by military families may impede continuity of care and delay referrals to complex epilepsy centers. The military discharged many adults with epilepsy, leaving them without long-term follow-up. This may contribute to lower responder rates of adult patients.

## Conclusion

Our data indicates that VNS is a well-tolerated long-term therapy for patients with DRE. Epilepsy duration of < 5 years and generalized epilepsy are independent predictors of a good response. Clinicians should incorporate VNS for the management of patients with drug-resistant generalized epilepsy, particularly in the early stages of the condition. This is a retrospective cohort study that is limited by a small sample size, recall bias, and lack of a control group. These results should be interpreted with caution. Future long-term RCTs with larger cohorts and comprehensive pre- and post-implantation assessments are needed to better assess these variables and their effects on VNS outcomes

## Data Availability

Raw data supporting the findings of this study are available on request from the corresponding author.

## Declarations

### Disclaimer

The views expressed in this article are those of the authors and do not reflect the official policy of the Department of Health Agency, Uniformed Services University of Health Sciences, or the U.S. government.
